# Mapping the resilience of chemosynthetic communities in hydrothermal vent fields

**DOI:** 10.1038/s41598-018-27596-7

**Published:** 2018-06-19

**Authors:** Kenta Suzuki, Katsuhiko Yoshida, Hiromi Watanabe, Hiroyuki Yamamoto

**Affiliations:** 1 Center for Environmental Biology and Ecosystem Studies, National Institute for Environmental Studies, 16-2, Onogawa, Tsukuba, Ibaraki 305-8506 Japan; 2 Research and Development (R&D) Center for Submarine Resources, Japan Agency for Marine-Earth Science and Technology, Natsushima-cho 2-15, Yokosuka, Kanagawa 237-0061 Japan

## Abstract

Hydrothermal vent fields are vulnerable to natural disturbances, such as volcanic activity, and are currently being considered as targets for mineral mining. Local vent communities are linked by pelagic larval dispersal and form regional metacommunities, nested within a number of biogeographic provinces. Larval supply depends on the connectivity of the dispersal networks, and affects recoverability of communities from disturbances. However, it is unclear how the dispersal networks contribute to recoverability of local communities. Here, we integrated a population dynamics model and estimation of large scale dispersal networks. By simulating disturbances to vent fields, we mapped recoverability of communities in 131 hydrothermal vent fields in the western Pacific Ocean. Our analysis showed substantial variation in recovery time due to variation in regional connectivity between known vent fields, and was not qualitatively affected by potential larval recruitment from unknown vent fields. In certain cases, simultaneous disturbance of a series of vent fields either delayed or wholly prevented recovery. Our approach is applicable to a dispersal network estimated from genetic diversity. Our method not only reveals distribution of recoverability of chemosynthetic communities in hydrothermal vent fields, but is also a practical tool for planning conservation strategies.

## Introduction

Chemosynthetic communities in hydrothermal vent fields (HVFs) demonstrate adaptations to extreme environments^1–5^ and provide various ecological services^6–9^. Local vent communities are linked by pelagic larval dispersal and form regional metacommunities, nested within a number of biogeographic provinces^10–12^. The incidence of disruptive natural disturbances to vent communities can range from several decades to several hundred years^13^. Faunal adaptations for colonising new vent fields are thus important aspects of the sustainability of these communities, especially since neighbouring vent fields are often separated by 10 s or 100 s of kilometres.

Recoverability, or resilience, refers to persistence of ecosystems in the face of natural or anthropogenic disturbances^14^. It can be quantified as the recovery time to the original state after disturbance^15^. For chemosynthetic communities in HVFs, observations on recovery from disruption caused by volcanic activities suggest that most of the diversity and biomass recovered within five years after the disturbance^16–19^, reviewed by Gollner *et al*.^20^. For example, total mega- and macrofaunal species richness at the vents in Juan de Fuca Ridge reached 75% of the pre-disturbance values three years after the 1998 eruption^18^, and 90% two years after the 1993 eruption^16^, representing about 30–60% of species from the larger regional species pool. At the East Pacific Rise (EPR), total mega- and macrofaunal species richness reached 69% of pre-disturbance values 4.6 years after the 1991 eruption^17^. After the EPR 2006 eruption, the recovery reached 55% for macrofaunal and 48% for meiofaunal species after 4 years^19^, with 39% of the macro and 42% of meiofaunal species returned. Recoverability varies significantly among communities in active vents, inactive vents, or within the vent periphery^20^. However, differences in recovery time among active vent fields are not well documented. Limited accessibility to HVFs (most of them are remote and in depth of more than 1,000 m) and rare opportunities to observe natural disturbances in less active volcanic areas (e.g., slow-spreading ridge systems and arc-backarc basin systems) hinders the ability of researchers to assess recoverability of chemosynthetic communities in the environment. To address this question, we integrated estimation of dispersal networks of HVFs with a differential equation model that describes the recovery of disturbed populations.

We used estimated larval dispersal between 131 HVFs in the western Pacific Ocean^12^. The estimates were based on a physical model of deep-ocean circulation that was validated through a deep-ocean profiling float experiment and considered temperature dependency of larval development that can control duration of pelagic larval stages. For example, larvae that use shallower depths can disperse further because of fast ocean currents and duration of the pelagic larval stage would be shortened when water temperature is higher^21^. Thus, dispersal distance will depend both on the speed of ocean currents and expected duration of larvae at a given depth. We assumed a dispersal depth of 1,000 m in our analysis based on the published data on the hydrographic structure of water columns obtained from observation and simulation models^22–24^. The biogeographic studies of larval dispersal at hydrothermal vents have a consensus on the transport mechanism by deep-sea advection and effluent layers^25,26^, where physicochemical parameters, physiographic features of a region and seafloor topography are recognized as possible barriers to dispersal^27^. The water mass below 1,000 m depth generally has stable physicochemical parameters, such as temperature and salinity, and may offer suitable conditions for larval survival in advection above the vent area and lateral transport in the effluent layer of the deep-sea. For example, there is a discontinuity in water temperature and salinity at 500–700 m in the Okinawa Trough, and at 1,000 m in the western and southern Pacific Ocean^28^. Moreover, two chemosynthetic communities found at a difference of more than 1,000 m depth had significant differences in their community structure^29^, suggesting the effect of environmental barriers (but see^30,31^ for recent findings).

The vent fields were separated into seven regions by grouping them in terms of their connectivity, i.e., each group had no interconnections (Table 1). The dispersal networks were implemented as a dispersal matrix A in the equation (1) below (see Methods).

1 $$\frac{{{\rm{d}}{\rm{x}}}_{{\rm{i}}}}{{\rm{d}}{\rm{t}}}=(1-\frac{{{\rm{x}}}_{{\rm{i}}}}{{{\rm{K}}}_{{\rm{i}}}})({{\rm{r}}{\rm{A}}}_{{\rm{i}}{\rm{i}}}{{\rm{x}}}_{{\rm{i}}}+{r{\rm{\Sigma }}}_{{\rm{i}}\ne {\rm{j}}}{{\rm{A}}}_{{\rm{i}}{\rm{j}}}{{\rm{K}}}_{{\rm{j}}}).$$

Here, K_i_ is the carrying capacity, (i.e., equilibrium population size of vent field i), r is the reproduction rate defined as the number of larvae that one individual produces per year and A_ij_ is the dispersal rate that a larva produced at vent field j will migrate into i per year, where A_ii_ corresponds to self-recruitment. Our model does not include the duration of pelagic larval phase as a parameter because it is included in the calculation of A^12,21^. We assumed that x_i_ is the population abundance of a species or group of species that share the same niche in vent field i. The species or group of species is assumed to distribute across all vent fields with sufficient abundance. This assumption is realistic because vent communities frequently have a dominant taxon that constitutes most of the biomass at a regional scale, e.g., *Bathymodiolus* mussels, *Shinkaia* squat lobsters and *Alviniconcha* gastropods^29,32,33^, although the dominance-diversity relationships may depend on the environmental conditions such as fluid-flux intensity and sediment types^34^. Amongst different regions however, it would be reasonable to interpret x_i_ as the abundance of different species or group of species that accounts for a similar proportion of biomass and having the same growth and dispersal characteristics. We do not directly consider the effect of disturbances on biological diversity because our model accounts for only one species. However, we expect that recoverability of these representative species would be a proxy for the recoverability of other infrequent community members, thus we regarded it as the recoverability of the community as a whole. For example, this is supported by observations of recovery after eruptions^16–20^ which showed a concordance of recovery in total organismal density and species richness. While we used the simplest model for our analysis, availability of more detailed data sets that include, for example, biomass, age structure or trophic interactions, would make more extensive models like those used for fishery stock assessments^35^ applicable and may provide more detailed insights.

**Table 1:** Profile of seven regions including the mean recovery time.

Region	Okinawa	Izu-Bonin	Mariana	Manus-Woodlark	Solomon	Kermadec	New Hebrides-North Fiji-Lau Tonga
Number of vent fields	7	4	16	15	4	8	74
Total number of links	32	9	105	67	8	41	2125
Mean in-degree (SD)	4.57 (1.51)	2.25 (0.96)	6.56 (1.36)	4.47 (2.07)	2.00 (1.15)	5.13 (1.36)	27.60 (13.76)
Mean self-recruitment 10^−6^ *larva/adults/year* (SD)	5194.57 (2688.58)	520.00 (273.81)	932.06 (346.50)	3501.80 (2450.62)	1108.50 (263.08)	1700.50 (808.89)	2074.86 (755.20)
Mean between vents recruitment 10^−6^ *larva/adults/year* (SD)	210.91 (3082.05)	232.92 (319.26)	297.26 (535.00)	633.06 (1488.82)	486.67 (624.21)	711.16 (1084.45)	449.63 (922.24)
Mean recovery time *year* (P value)	5.76 (0.11)	138.42 (0.00)	23.86 (0.19)	27.78 (0.11)	49.17 (0.07)	16.18 (0.40)	6.09 (0.00)

Except for the mean recovery time, values in the table were calculated from the dispersal matrix in Table S5. Here, “Mean in-degree” is the mean number of incoming links per vent fields within a region, “Mean self-recruitment” is the mean of self-recruitment (A_ii_) within a region and “Mean between vents recruitment” is the mean of A_*ij*_ (i ≠ j) within a region. “Mean self-recruitment” and “Mean between vents recruitment” is shown as multiplied by 10^6^ to facilitate understanding.

By considering previous observations^16–20^, we defined τ_i_ as the mean recovery time of a vent field i for the ensemble of various spatial distributions of K_i_, where recovery time is the time required for x_i_ to recover 75% of its original abundance (equal to K_i_ by definition) after it was temporarily reduced to zero. To calculate τ_i_, we assumed that K_i_ is assigned from a uniform distribution P_K_ = 2^q^K_0_ where q is a uniform distribution between −1 to 1 and K_0_ = 10000, and all vent fields except for i are in equilibrium. However, the following results are independent of K_i_ if K_i_ values are identical across vent fields (equation (4)). Furthermore, they can be referred to as the mean recovery time in the ensemble of potential spatial heterogeneity in K_i_ as long as K_i_ of all vent fields follows the same probability distribution (see Methods). For simplicity of notation, in what follows, we regard the set of τ_i_s for all vents fields as {τ_i_}. Here, we do not consider the effect of time on K_i_. However, in nature, K_i_ will not be fixed in time. Our result may underestimate recovery time if frequency of a species is highly constrained by the amount of suitable habitat that increases slowly along with the re-establishment of vent fields after disturbances. For example, the loss and recovery of mussel beds may restrict abundance of associated small invertebrate species^36^.

## Results and Discussion

**Figure 1:**
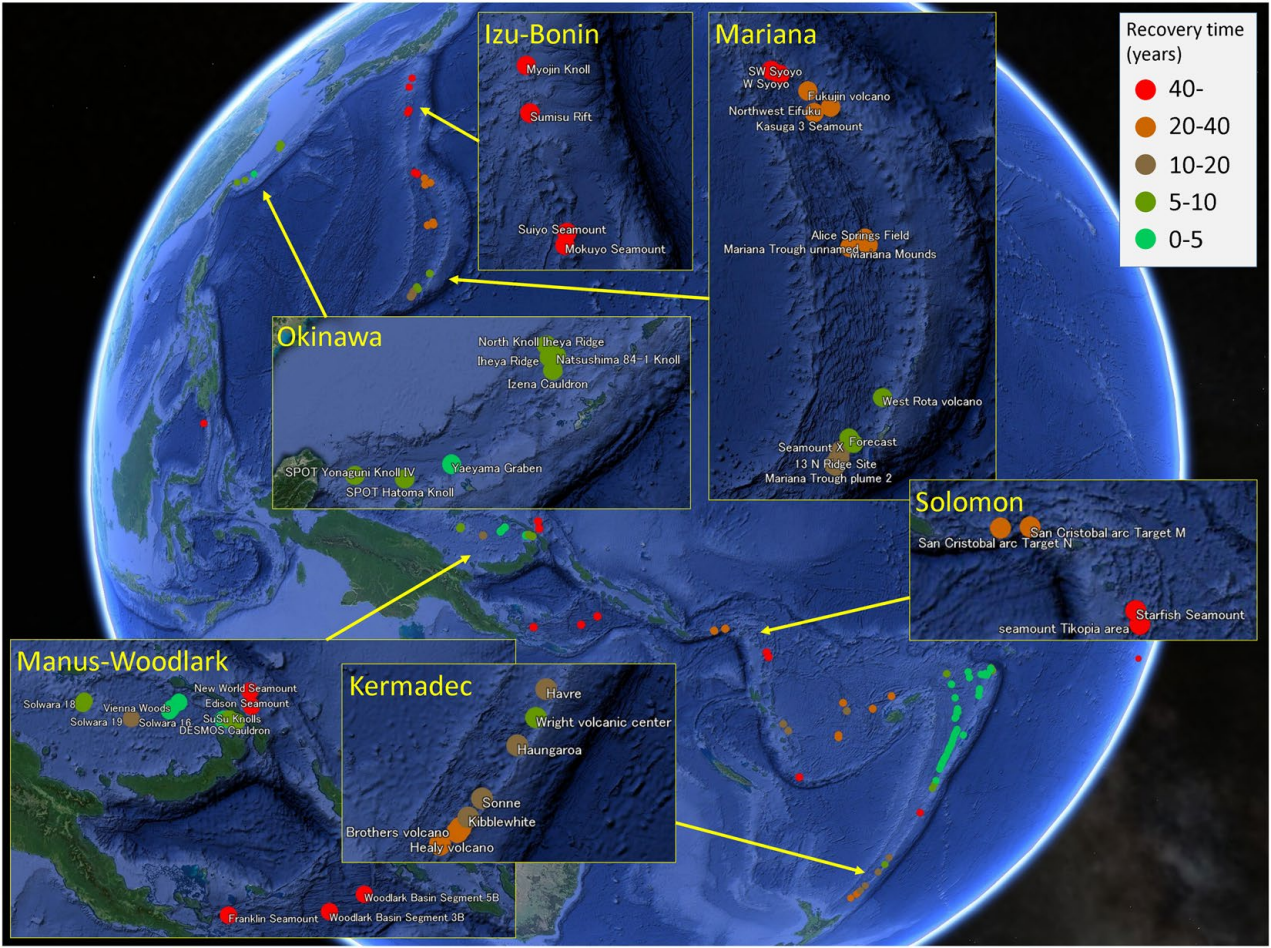
Recovery time of communities in HVFs in western Pacific Ocean. See Supplementary Fig. S1 for New Hebrides-Lau Tonga-North Fiji. The map was generated from digital information available at Google Earth Pro v7.3.0.3832 (https://www.google.com/intl/en/earth/; Map data: Google Earth, Image Landsat/Copernicus, Data SIO, NOAA, U.S. Navy, NGA, GEBCO).

We obtained the spatial distribution of τ_i_ as in Fig. 1. To be consistent with previous observations^16–20^, we set r = 17.4 so that the median of {τ_i_} was standardised to five years (Supplementary Fig. S2). Hence, we used five years as a reference representing a standard time scale of recovery of HVFs from large disturbances. Mean τ_i_ in Izu-Bonin was 131 years (Table 1) and the shortest τ_i_ (Mokuyo Seamount) was 56 years (Fig. 1; see Supplementary Table S1 for further information). Mean τ_i_ in Manus-Woodlark, Solomon and Mariana was also longer than 20 years. These regions contained multiple HVFs with τ_i_ longer than 40 years: W Syoyo and SW Syoyo in Mariana, New World Seamount, Edison Seamount, Woodlark Basin Segment 5B, Woodlark Basin Segment 3B and Franklin Seamount in Manus-Woodlark, Starfish Seamount and Tikopia Area in Solomon. *P* values of the τ_i_ were low, suggesting region specific factors were determinants of recovery time. There was a large contrast between regions with high recoverability (Okinawa and New Hebrides-North Fiji-Lau Tonga) and regions with low recoverability (Izu-Bonin, Manus-Woodlark, Mariana and Solomon; Table 1; see Methods). The inequality in recovery time was dependent on the interplay between the ocean circulation and the spatial distribution of HVFs in each region. Within some regions (Mariana, Manus-Woodlark and NewHebrides-NorthFiji-LauTonga), we found a positive correlation between τ_i_ and the distance of HVFs from the mean (see Supporting Table S2). Hence, the recovery time of a HVF would be biased depending on the region to which it belongs and occasionally also on its locality within the region. We did not include Eva, Kaiwo Barat and Teahitia Vents in our results because these HVFs had no known incoming links and thus were unable to recover based on our assumptions. It may be unrealistic to judge that communities in these HVFs cannot completely recover from disturbances because there would be overlap of species between communities in active vent fields and other environments, such as inactive vents or along the vent periphery^37,38^. Some opportunistic/non-endemic species could colonise a recently disturbed vent field through adult migration rather than being limited by larval dispersal^6^. This will contribute to the recovery of communities in these HVFs (and of course, also affect recovery of communities in HVFs having incoming links). However, we did not consider this because the interrelationship between larval dispersal and adult migration for population recoverability is an issue of complexity within the local community.

**Figure 2:**
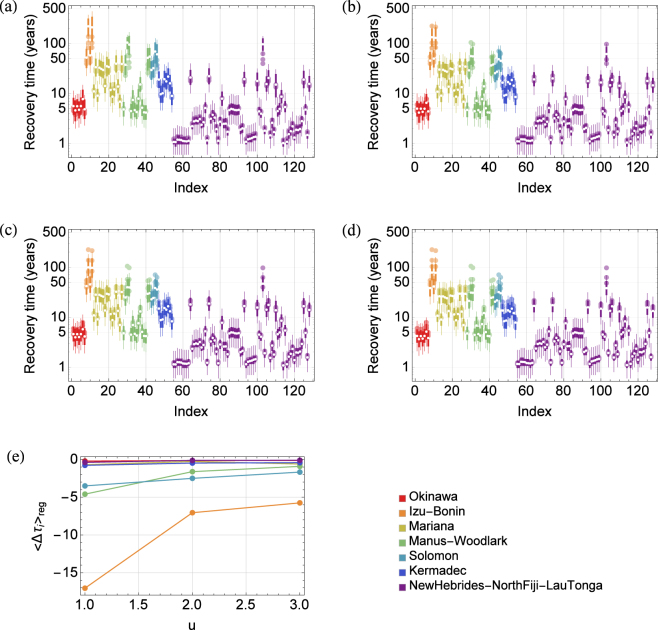
Mean and variation of recovery time including the effect of unknown vent fields. (**a**) Mean recovery time (τ_i_; white points) and 95% CI (thick and thin lines) calculated by the model without effect of unknown vent fields (equation (1), or equation (2) with u = 0). Here, thick and thin lines indicate the 95% CI when K_i_ was fixed at the mean of P_K_ (i.e., K_i_ = 10,000) and when it was also assigned from P_K_, respectively. (**b**–**d**) The same result for u = 1, 2, 3, respectively. In (**a**–**d**), points with light colors indicate τ_i_ for other u values. For u = 0, 1, 2, 3, we set r = 17.4, 16.7, 16, 15.6, respectively, to keep median of τ_i_s as five years. (**e**) The reduction of mean recovery time within a region, defined as ${\langle {{\rm{\Delta }}{\rm{\tau }}}_{{\rm{i}}}({\rm{u}}+1)\rangle }_{{\rm{reg}}}={\langle {{\rm{\tau }}}_{{\rm{i}}}({\rm{u}}+1)\rangle }_{{\rm{reg}}}-{\langle {{\rm{\tau }}}_{{\rm{i}}}({\rm{u}})\rangle }_{{\rm{reg}}}$ is shown. Here, 〈·〉_reg_ is used to emphasize that it is the mean of recovery time within a region.

95% CI of recovery time (thick and thin lines) is shown in Fig. 2a with τ_i_ (shown in white points). While τ_i_ was mostly explained by the total A_ij_ (ΣA_ij_) to HVFs (Supporting Fig. S3), there were two factors that affected 95% CI of recovery time. One was K of disturbed HVF (K_i_) and the other was that of other HVFs (K_j_ values). Figure 2a shows the 95% CI when K_i_ was fixed at the mean of P_K_ (i.e., K_i_ = 10000; thick lines) and when it was also assigned from P_K_ (thin lines). Thus, Fig. 2a illustrates relative contribution of K_i_ and K_j_ values to the 95% CI as the length of thick and thin lines, respectively. Because the 95% CI was the result of the ensemble of virtual distribution of K values, these lines represent uncertainty in recovery time when we do not have information on both K_i_ and K_j_ values (thin lines) and when we can specify K_i_ (thick lines). Supplementary Fig. S4 shows how much uncertainty can be reduced by specifying K_i_. The uncertainty in recovery time decreased substantially when ΣA_ij_ was large because HVFs also had large in-degree (Supplementary Fig. S5), and the law of large numbers reduced the effect of variation of K_j_ values (i.e., the variation of recovery time was explained by the variation of K_i_ itself). It is also worth noting that HVFs in Okinawa and several HVFs in Manus-Woodlark (e.g., DESMOS Cauldron, PACMANUS field and Solwara 13, etc.) had larger uncertainty for K_j_s than HVFs in New Hebrides-North Fiji-Lau Tonga having similar ΣA_ij_ (e.g., Mata Fa, Mata Fitu and Mata Ono, etc.) (Fig. 2a), which was also explained by the difference of in-degree among these HVFs (Supplementary Fig. S5).

Several unknown HVFs may exist in each region and provide larvae to known HVFs. The effect of unknown HVFs can be introduced as in equation (2). Here, we introduced a parameter u as the number of unknown HVFs in a region, and set r so that median of {τ_i_} was kept identical to five years for each u (see legend of Fig. 2).

Addition of unknown HVFs generally reduced both mean and variation of recovery time (Figs 2, S4 and Supplementary Table S1). In particular, unknown fields had a large effect in the regions with large τ_i_, such as Izu-Bonin, Solomon and Manus-Woodlark (Fig. 2e). However, in Mariana, the effect of unknown HVFs was similar to that of Okinawa or Kermadec despite a higher mean τ_i_ in these region (Table 1). This is due to the fact that Mariana has many HVFs with high inter-connectivity (in-degree was 6.56, the second highest among all regions), but a low dispersal rate per link (mean between vent recruitment was 232.91 10^−6^ *larvae/adults/year*, the second lowest among all regions). Hence, addition of several unknown HVFs had only a small effect on τ_i_ in Mariana. Mean τ_i_ of Izu-Bonin, Manus-Woodlark and Solomon would be largely overestimated considering the potential effect from unknown HVFs. However, their *P* values still suggested inequality in recoverability among regions (Supplementary Table S3). Hence, presence of unknown HVFs would not qualitatively change the imbalance of recovery time.

On-going development of deep-sea resource mining technologies^38–40^ raises the possibility that natural and anthropogenic factors will simultaneously disturb multiple HVFs in a region. We evaluated recovery time for disturbance on multiple HVFs as τ_C_, where C = {i, j, …} is a possible combination of disturbed HVFs in a region. We tested all combinations if the total number of combinations was less than 2,000, and, if the total number exceeded 2,000, we randomly selected 2,000 combinations without duplication. The procedure to calculate recovery time is the same as previous analyses except the abundances of populations in HVFs included in C were simultaneously reduced to zero. τ_C_ is the largest recovery time of HVFs included in C, i.e., τ_c_ = max({τ_i_}_i∈c_). For simplicity, we did not consider variation of K in this analysis and assumed K = 10,000 for all HVFs, i.e., we focused on the effect of disturbances on the mean recovery time.

**Figure 3:**
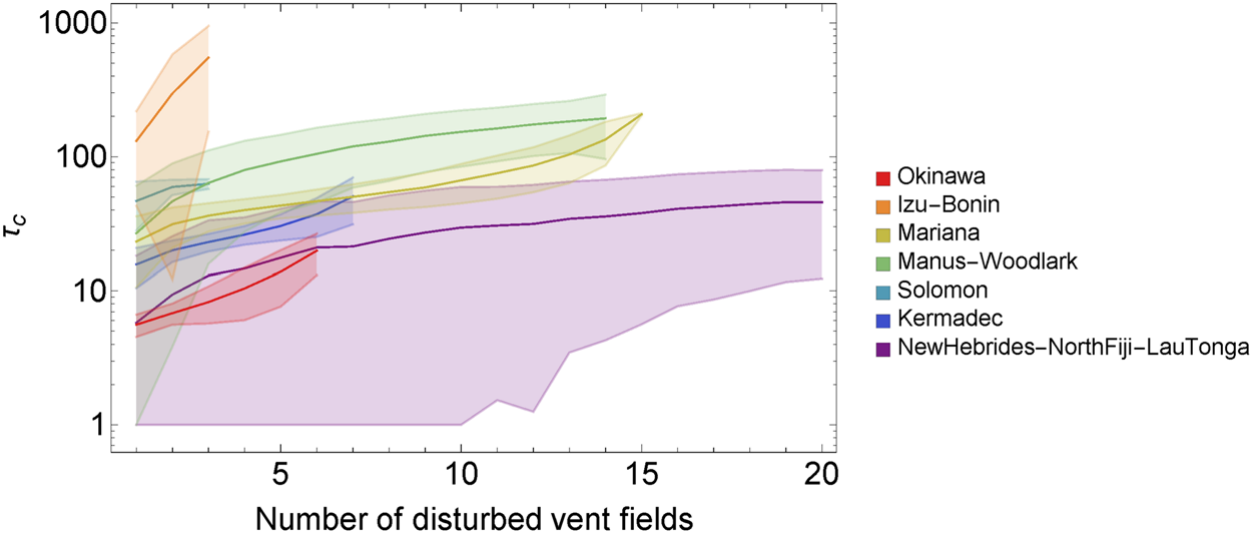
Result of simultaneous disturbances to multiple vent fields. To calculate τ_C_, we set u = 0 and r = 17.4. Lines and colored area indicate the mean and 95% CI of τ_C_, respectively. Combinations that include unrecoverable cases have been removed and are shown in Fig. S3.

In Fig. 3, τ_c_s are shown as a function of the number of simultaneously disturbed HVFs with the 95% CI. The mean recovery time monotonously increases with the number of disturbed HVFs, while the pattern of the increase is different depending on differences in connectivity of each region. We found that some combinations extremely delay recovery compared to others. For example, when more than three HVFs were disturbed in Okinawa, τ_C_ was larger than 20 years if all of Hatoma Knoll, Dai-Yon Yonaguni Knoll and Irabu Knoll were included in the disturbance (this is shown in panel (a) of Supplementary Fig. S6 where a point indicating recovery time longer than 20 years first appears when three HVFs are simultaneously disturbed), while that of other combinations were smaller than 10 years. This is clear from the dispersal matrix in this region (Supplementary Table S4) showing that if all three HVFs are disturbed, recovery will depend on dispersal from Iheya Ridge to Hatoma Knoll and Dai-Yon Yonaguni Knoll, and dispersal via these links is more than 10 times smaller than the mean between vent dispersal in this region (Table 1). In addition, Mariana, Manus-Woodlark and Solomon had combinations that prevent recovery (Supporting Fig. S7). In these cases, no larval supply from the undisturbed HVFs to the disturbed HVFs remained after the disturbance. For example, in Manus-Woodlark, no larval supply remained if Woodlark Basin Segment 5B, Woodlark Basin Segment 3B and Franklin Seamount were simultaneously disturbed. Thus, when disturbance to multiple HVFs is considered, inclusion of some HVFs can either slow down or completely prevent recovery. Further investigation on this observation would benefit by incorporating network analysis, e.g. use of centrality measure^41^ to distinguish source and sink HVFs, and would provide relevant insight for the application of our approach to management strategy planning. This application would be further strengthened if knowledge on the frequency of natural disturbances (e.g., cycles of volcanic and hydrothermal activities) in each region could be integrated.

A method using genetic diversity to estimate the dispersal matrix for a species distributed across multiple HVFs has been proposed^42,43^. Using the dispersal matrix of a galatheid crab, *Shinkaia crosnieri*, in Okinawa Trough (Watanabe *et al*. unpublished data), we found some consistency between the dispersal matrix estimated from the bio-physical model^12^ and the matrix estimated from genetic diversity in terms of recovery time (supplementary material). It is worth noting that the latter does not explicitly include information about ocean circulation.

To date, 700 HVFs have been found worldwide^44^. 57% (399) of them are located on the mid-ocean ridge system and the rest are on arc-backarc systems. 88% (249 among 283) of arc-backarc systems are distributed in the Pacific Ocean, of which this study included 52%. In future studies, researchers will reveal dispersal networks in a broader region of the world ocean and range of taxonomy. The coverage of this analysis can be extended provided data are available. Our approach will allow us to map the resilience of different species, enable comparative studies on the relationship between resilience and species traits and, when the model is modified to multispecies systems, it will further help us to understand community dynamics, such as succession. Thus, we think that this study is also an important step towards understanding the processes that form and sustain communities in HVFs. Conservation strategies for deep-sea ecosystems may include creation of new habitats as well as design of ecologically protected areas^38,45–47^. Our methodology would reasonably direct these efforts by predicting the outcome of various strategies in any region worldwide. However, it should be mentioned that there is still a relative lack of empirical data from disturbance-recovery studies that can support our results. All previous studies have been carried out on either the Juan De Fuca Ridge^16,18^ or the East Pacific Rise^17,19^. Because both are in a fast spreading ridge where biological communities are frequently disturbed (e.g., ~15 years^48^), it would be controversial whether these results represent recovery of HVFs in slow spreading ridges or arc-backarc basins where disturbance to communities are relatively infrequent^20^. However, our results still suggest substantial difference of recovery time among HVFs, which can span two orders of magnitude, highlighting the importance of understanding connectivity among HVFs to assess their recoverability.

## Methods

### Population dynamics

We assumed that populations in a vent field are supported by recruitment from the same vent field (self-recruitment) as well as recruitment from other vent fields. For equation (1), we assumed that K_i_ is determined by the total amount of resource supply in vent field i, and limits maximum population size. rA_ii_x_i_ represents the larval supply via self-recruitment per year and rΣ_i≠j_A_ij_K_j_ is the total larval supply from other vent fields per year, assuming that other vent fields are in equilibrium (x_j_ = K_j_).

### Estimation of the effect of unknown vent fields

To estimate the effect from unknown vent fields, we assumed that each unknown vent field supplies larvae to all known vent fields in the region to which it belongs and the amount of larval supply per each link is identical to the mean between patch recruitment in the region (${\epsilon }_{0}$; Table 1). By adding the effect from unknown vent fields s as ${\epsilon }_{0}{\rm{u}}$ to equation (1), we obtain,

2 $$\frac{{{\rm{d}}{\rm{x}}}_{{\rm{i}}}}{{\rm{d}}{\rm{t}}}=(1-\frac{{{\rm{x}}}_{{\rm{i}}}}{{\rm{K}}})({{\rm{r}}{\rm{A}}}_{{\rm{i}}{\rm{i}}}{{\rm{x}}}_{{\rm{i}}}+{r{\rm{\Sigma }}}_{{\rm{i}}\ne {\rm{j}}}{{\rm{A}}}_{{\rm{i}}{\rm{j}}}{{\rm{K}}}_{{\rm{j}}}+{\epsilon }_{0}{\rm{u}}),$$

where, ${\epsilon }_{0}$ is the mean between patch recruitment in the region that i belongs to, and u is the number of unknown vent fields.

### Analytical calculation of recovery time

Recovery time can be simply obtained by numerically solving equations with appropriate numerical integration scheme such as the Runge-Kutta method. However, it is more convenient to use their analytical solution if only one vent field is disturbed. For example, for equation (1), we obtain,

3 $${\rm{t}}({{\rm{x}}}_{{\rm{i}}})=\frac{1}{{\rm{r}}({{\rm{A}}}_{{\rm{i}}{\rm{i}}}+{{\rm{K}}}_{{\rm{i}}}^{-1}{{\rm{\Sigma }}}_{{\rm{i}}\ne {\rm{j}}}{{\rm{A}}}_{{\rm{i}}{\rm{j}}}{{\rm{K}}}_{{\rm{j}}})}\,{\rm{l}}{\rm{o}}{\rm{g}}\,\frac{{\rm{r}}({{\rm{A}}}_{{\rm{i}}{\rm{i}}}{{\rm{x}}}_{{\rm{i}}}+{{\rm{\Sigma }}}_{{\rm{i}}\ne {\rm{j}}}{{\rm{A}}}_{{\rm{i}}{\rm{j}}}{{\rm{K}}}_{{\rm{j}}})}{{{\rm{K}}}_{{\rm{i}}}-{{\rm{x}}}_{{\rm{i}}}}.$$

Moreover, if K_i_ is identical across vent fields, i.e. K_i_ = K, by substituting x_i_ = αK, we obtain,

4 $${{\rm{t}}}_{{\rm{i}}}=\frac{1}{{\rm{r}}({{\rm{A}}}_{{\rm{i}}{\rm{i}}}+{{\rm{\Sigma }}}_{{\rm{i}}\ne {\rm{j}}}{{\rm{A}}}_{{\rm{i}}{\rm{j}}})}\,{\rm{l}}{\rm{o}}{\rm{g}}\,\frac{{\rm{r}}({{\rm{A}}}_{{\rm{i}}{\rm{i}}}\alpha +{{\rm{\Sigma }}}_{{\rm{i}}\ne {\rm{j}}}{A}_{{\rm{i}}{\rm{j}}})}{1-\alpha }.$$

Here, α is the abundance criteria (ratio) to judge recovery, which we set as 0.75 in this paper. Equation (4) shows that recovery time is independent of K if it is identical across vent fields.

### Calculation of P values for mean recovery time

*P* values for the mean recovery time of each region were obtained by using {τ_i_} as the empirical distribution. Here, for each region, we simply repeated random sampling from the empirical distribution up to the number of vent fields in the region, then calculated the mean recovery time. This procedure was repeated 10,000 times providing 10,000 bootstrap samples for each region from which we evaluated the *P* value of the mean recovery time calculated from actual τ_i_ values.

### Spatial heterogeneity in population size

If carrying capacity K_i_ of all vent fields follows the same probability distribution Φ, equation (4) holds for the mean of Φ, μ_K_, i.e., for μ_K_, recovery time is independent of Φ. For convenience of explanation, we express κ^k^ = {K_i_}_i=1,…,N_ as the kth distribution of K_i_s among its possible spatial distribution under K_i_ ~ Φ and ${\{\overline{{{\rm{\tau }}}_{{\rm{i}}}}\}}_{{\rm{i}}=1,\ldots ,{\rm{N}}}$ is the recovery time when K_i_ is identical across all vent fields. The implication of the above statement on the independence of recovery time from Φ will become clear if we consider an ensemble of {τ_i_}_i=1,…,N_ for κ^k^s as {T_i_}_i=1,…,N_, where ${{\rm{T}}}_{{\rm{i}}}={\{{{\rm{\tau }}}_{{\rm{i}}}^{({\rm{k}})}\}}_{{\rm{k}}=1,\ldots ,{\rm{M}}}$. Here, because the mean of K_i_ over k is μ_K_ for all vent fields, the mean of {T_i_}_i=1,…,N_, ${\{{\rm{\langle }}{{\rm{T}}}_{{\rm{i}}}{\rm{\rangle }}\}}_{{\rm{i}}=1,\ldots ,{\rm{N}}}={\{{{\rm{\Sigma }}}_{{\rm{k}}}{{\rm{\tau }}}_{{\rm{i}}}^{({\rm{k}})}/M\}}_{{\rm{i}}=1,\ldots ,{\rm{N}}}\,$, becomes identical to ${\{\overline{{{\rm{\tau }}}_{{\rm{i}}}}\}}_{{\rm{i}}=1,\ldots ,{\rm{N}}}$, which is independent of μ_K_ and even Φ. Hence, {T_i_}_i=1,…,N_ is identical to ${\{\overline{{{\rm{\tau }}}_{{\rm{i}}}}\}}_{{\rm{i}}=1,\ldots ,{\rm{N}}}$. There is no model that reasonably explains different probability distributions of K_i_ in different regions as well as provides information to specify K_i_ of each vent field. As a base line expectation, the best approach to implement spatial heterogeneity in K_i_ is to consider the ensemble. Hence, in this paper, we identify {〈T_i_〉}_i=1,…,N_ with ${\{\overline{{{\rm{\tau }}}_{{\rm{i}}}}\}}_{{\rm{i}}=1,\ldots ,{\rm{N}}}$ (we simply denoted $\overline{{{\rm{\tau }}}_{{\rm{i}}}}$ as τ_i_ in other parts of this paper).

## Electronic supplementary material

Supplementary information

supplementary Table S1

supplementary Table S5
