# Supplementary material for: Mapping the resilience of chemosynthetic communities in hydrothermal vent fields
Source: Sci Rep. 2018 Jun 19;8:9364. doi: 10.1038/s41598-018-27596-7 (PMC6008444; doi:10.1038/s41598-018-27596-7)
Supplement: Supplementary file 1 — Supplementary information [file 41598_2018_27596_MOESM1_ESM.pdf]

Supplementary Information

# Mapping the resilience of chemosynthetic communities in hydrothermal vent fields

Kenta Suzuki[1], Katsuhiko Yoshida[1], Hiromi Watanabe[2] and Hiroyuki Yamamoto[2]

1. Center for Environmental Biology and Ecosystem Studies, National Institute for Environmental Studies. 16-2, Onogawa, Tsukuba, Ibaraki, 305-8506, Japan.

2. Research and Development (R&D) Center for Submarine Resources, Japan Agency for Marine-Earth Science and Technology, Natsushima-cho 2-15, Yokosuka, Kanagawa 237-0061, Japan.

Corresponding author: Kenta Suzuki

Tel.: +81-029-850-2747

E-mail: suzuki.kenta@nies.go.jp

## Estimation of recovery time from genetic diversity

To implement a dispersal matrix estimated from local genetic diversity, some modifications to equation (1) were required because the elements of the dispersal matrix are estimated as the number of individuals arriving from vent fields j to i per generation time. Here, generation time, g, is defined as the time interval from birth to maturity. The equation is obtained as,

$dx_i/dt=(1-x_i/K_i)((Rx_i)/g+(\Sigma_{(j\neq i)} A_{ji})/g)$. (3)

Here, R represents the reproduction rate of one individual per generation time. In equation (3), we defined $A_{ii}/R$ as $K_i$ assuming that, among all larvae produced per year, the ratio of larvae that contributes to self-recruitment is identical across vent fields. $Rx_i/g$ represents the larval supply via self-recruitment per year, where R/g is the reproduction rate per year. Similarly, $\Sigma_{(j\neq i)} A_{ji}/g$ represents the total larval supply from other vent fields per year.

To compare the dispersal matrix estimated from the bio-physical model and the matrix estimated from genetic diversity, we used the dispersal matrix of a galatheid crab, Shinkaia crosnieri, (Watanabe et al. unpublished data). The galatheid crab is one of the dominant species in Okinawa region (Okinawa Trough; Fujikura et al. 2002). We selected parameters of equation (3) so that the mean $\tau_i$ became equal to 5.7 years (in Table 1): the condition was R=1/12.0 if we set g=1. The standard deviation of the recovery time of the galatheid crab population (Table S6) was 1.139, which was close to the standard deviation of recovery time calculated from Mitari et al.'s (2016) result, 1.063. Moreover, recovery time of Dai-Yon Yonaguni Knoll which appears in both data was second from the bottom (6.7 years) for the galatheid crab population, and first from the bottom (7.5 years) when it was calculated from Mitarai et al.'s (2016) result. Hence, the two dispersal matrices for Okinawa region have the same tendency for larval dispersal although the latter does not use explicit information about ocean circulation.

Reference

Fujikura, K., Hashimoto, J., & Okutani, T. (2002). Estimated population densities of megafauna in two chemosynthesisbased communities: a cold seep in Sagami Bay and a hydrothermal vent in the Okinawa Trough. Benthos Research, 57(1), 21-30.

Supplementary Figures

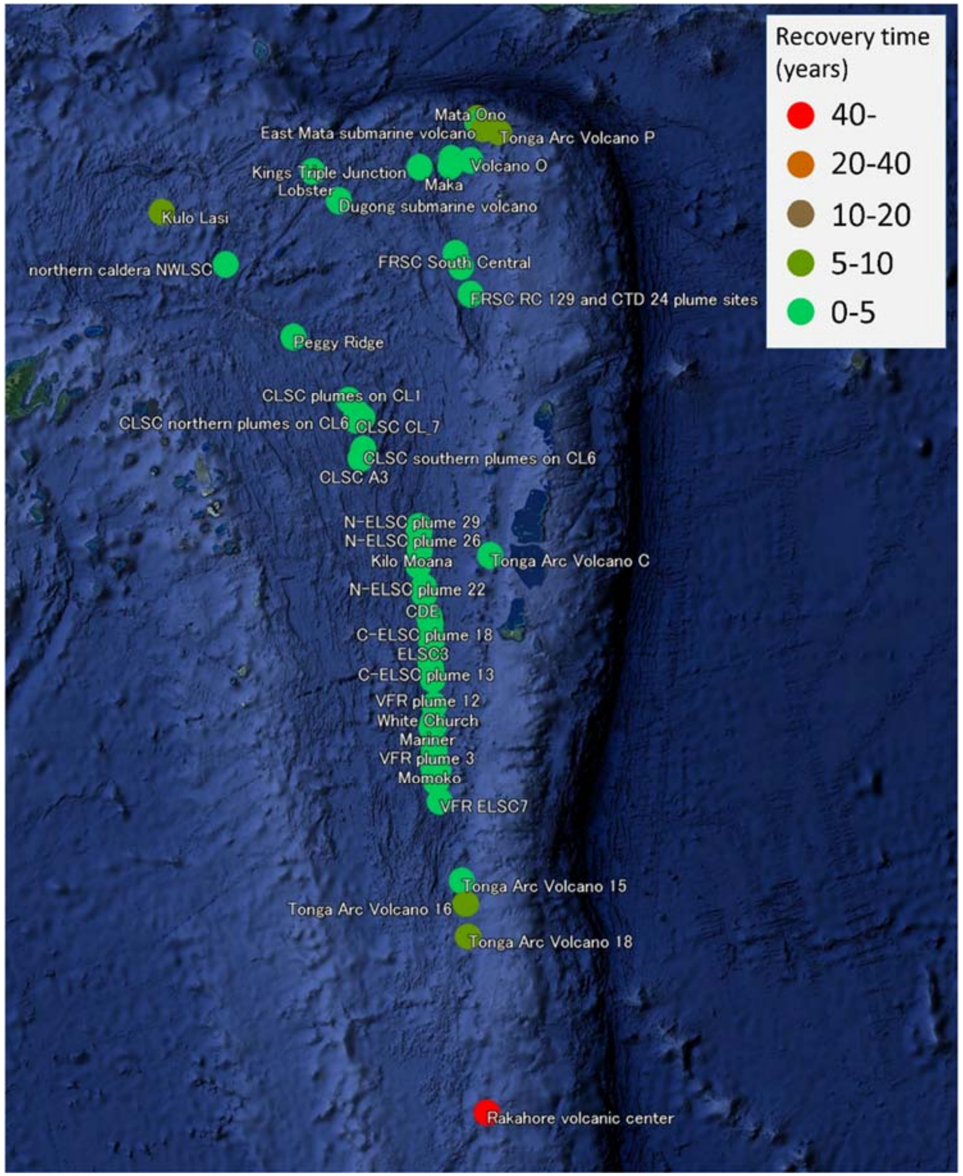


Figure S1. Recovery time of communities in hydrothermal vent fields in New Hebrides-Lau Tonga-North Fiji. The map was generated from digital information available at Google Earth Pro v7.3.0.3832 (https://www.google.com/intl/en/earth/; Map data: Google Earth, Image Landsat/Copernicus, Data SIO, NOAA, U.S. Navy, NGA, GEBCO).

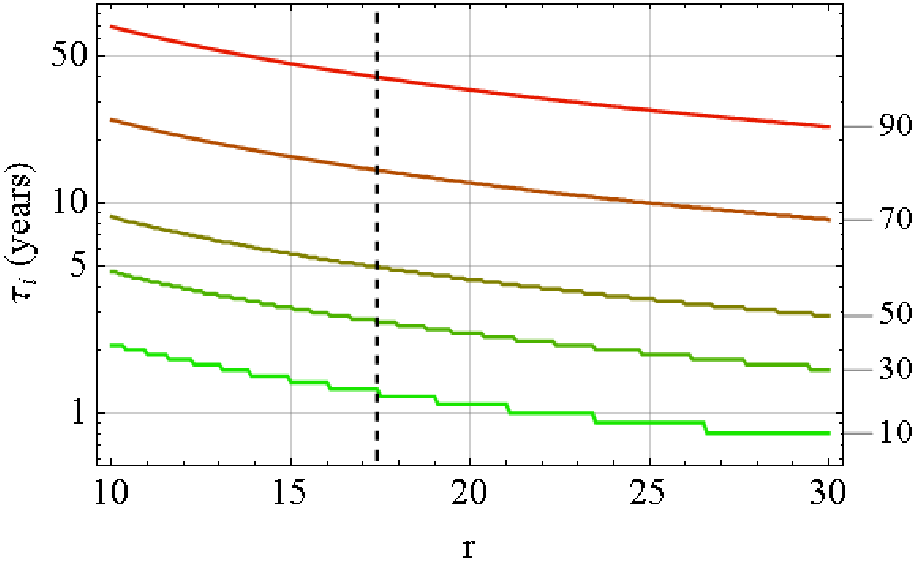


Figure S2. Cumulative distribution of $\tau_i$ as a function of $r$. Percentile values (10-90%) are indicated by solid lines. Dashed line indicates $r = 17.4$ at which the median of $\{\tau_i\}$ becomes five years.

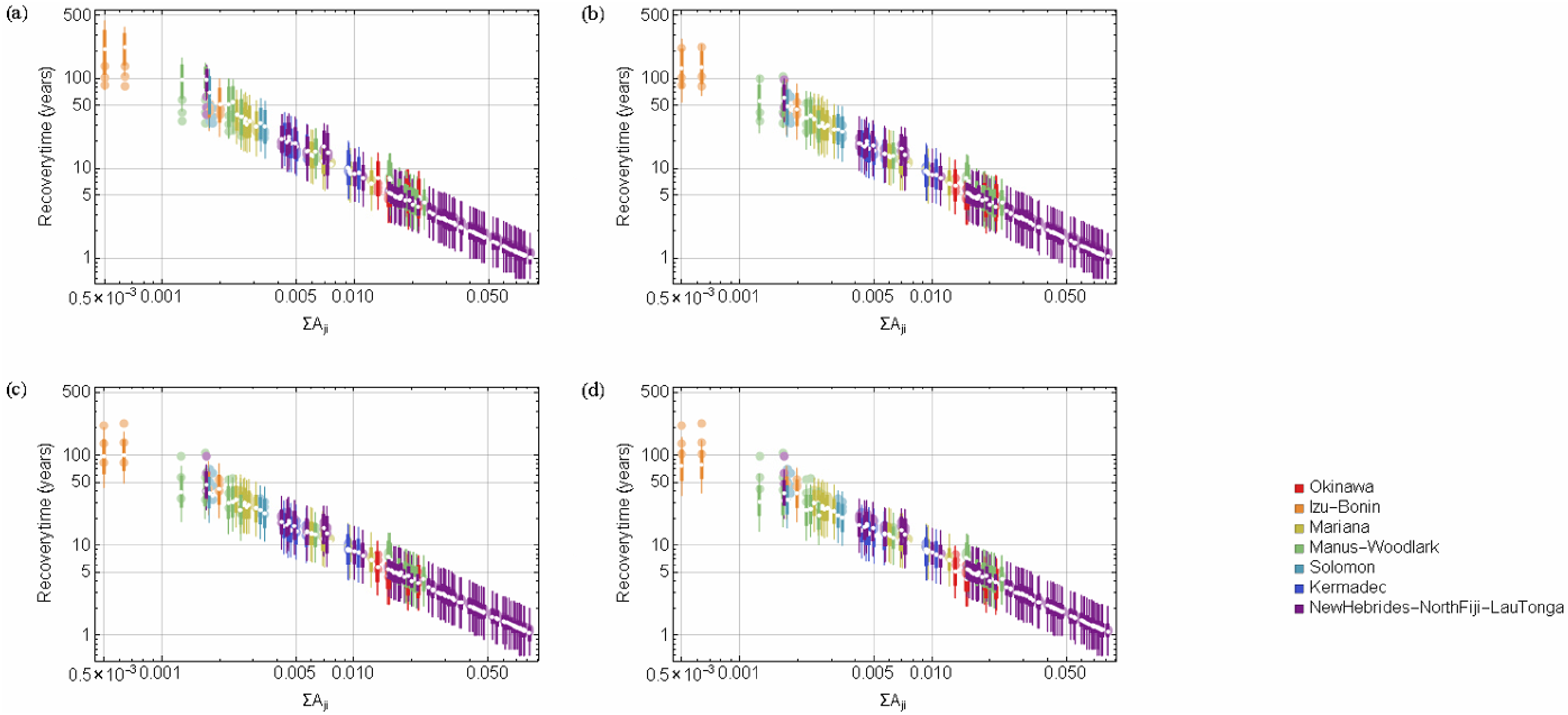


Figure S3. Mean and variation of recovery time as a function of total $A_{ji}$ ($\Sigma A_{ji}$). (a-d) Mean recovery time ($\tau_i$; white points) and 95% CI (thick and thin lines) calculated by the model with u=0,1,2,3, respectively. Here, thick and thin lines indicate the 95% CI when $K_i$ was fixed at the mean of $P_K$ (i.e., $K_i$=10,000) and when it was also assigned from $P_K$, respectively. In (a-d), points with light colors indicate $\tau_i$ for other u values. For u=0,1,2,3, we set r=17.4,16.7,16,15.6, respectively, to keep median of {$\tau_i$} as five years.

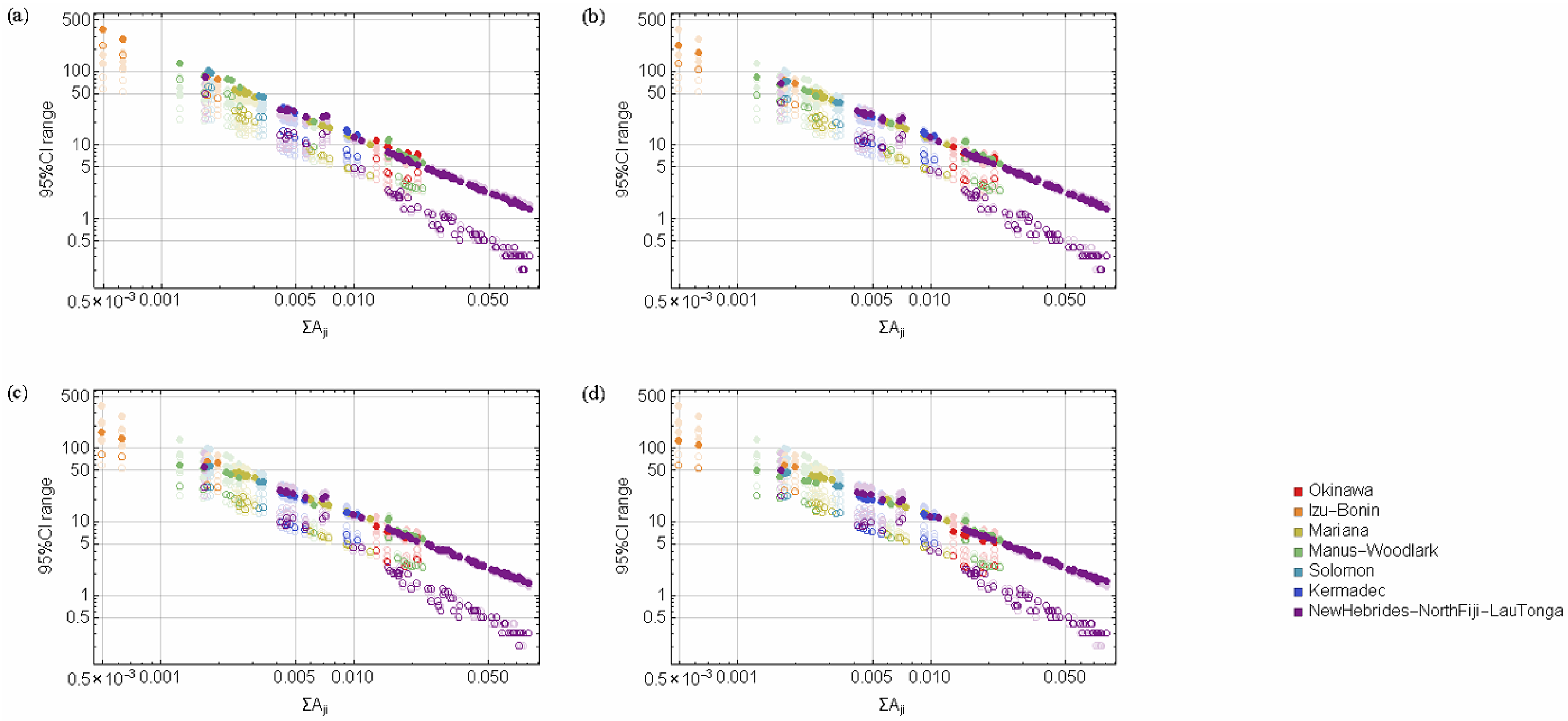


Figure S4. 95% CI range of recovery time as a function of total $A_{ji}$ ($\Sigma A_{ji}$). (a-d) 95% CI range calculated by the model with u=0,1,2,3, respectively. Points and circles indicate the 95% CI when $K_i$ was fixed at the mean of $P_K$ (i.e., $K_i$=10,000) and when it was also assigned from $P_K$, respectively. In (a-d), points and circles with light colors indicate 95% CI range calculated for other u values.

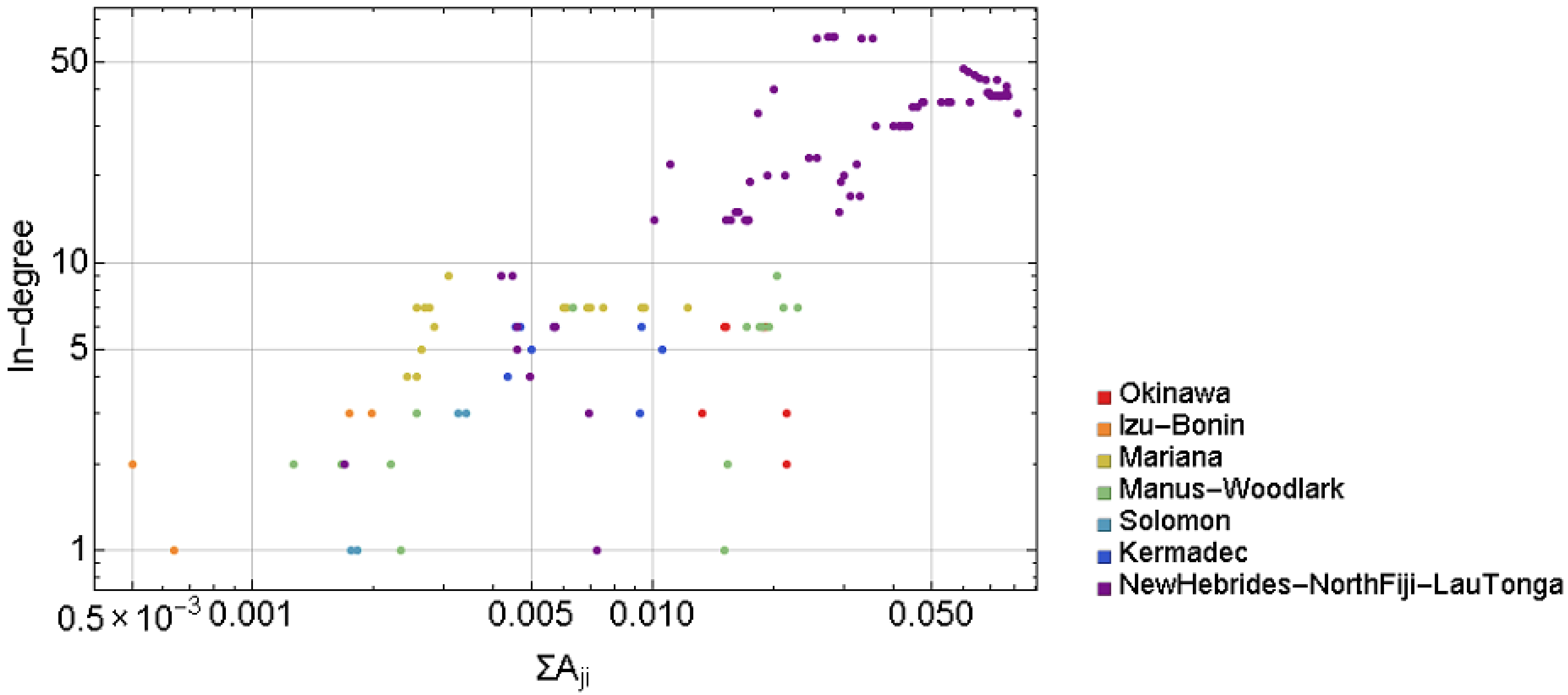


Figure S5. In-degree as a function of total Aji ($\Sigma A_{ji}$)

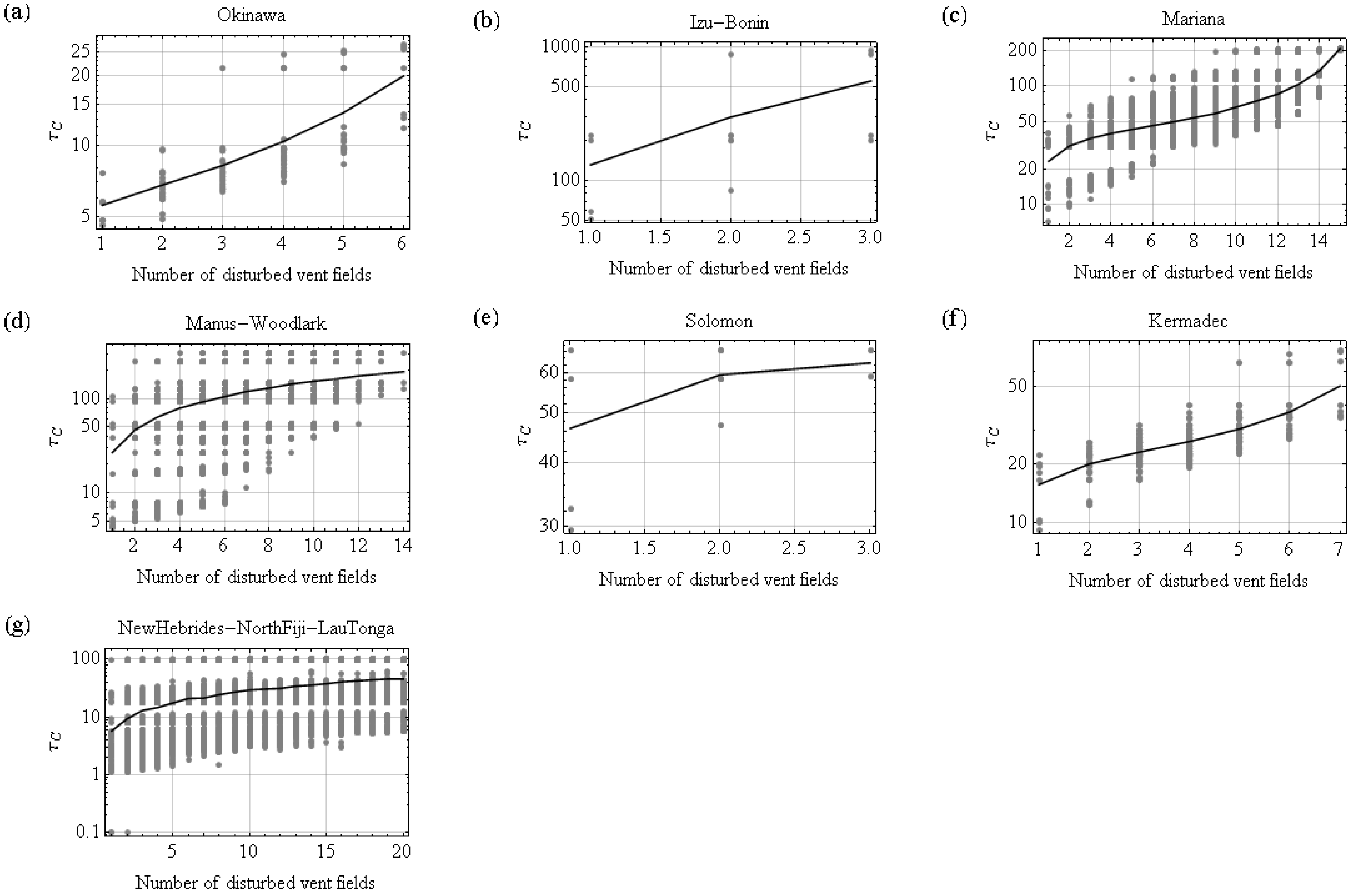


Figure S6. Result of simultaneous disturbances to multiple vent fields. To calculate $\tau_C$, we set $u = 0$ and r=17.4. Points in figure show $\tau_C$ of a combination and lines

indicates the mean. Combinations that include unrecoverable cases have been removed and are shown in figure S3.

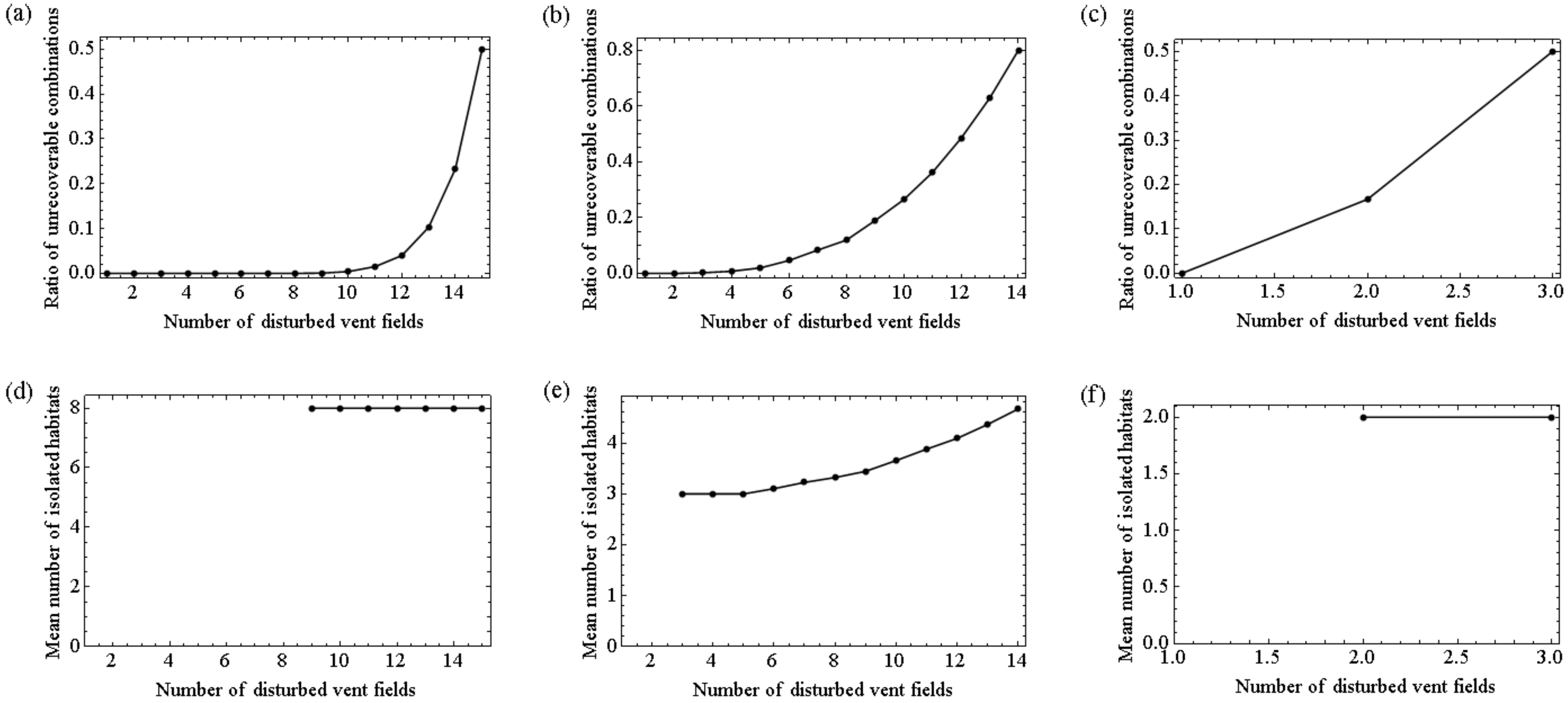


Figure S7. Combination of disturbances that prevent recovery. The unrecoverable combinations were found in Mariana (a,d), Manus-Woodlark (b,e), Solomon (d,f). (a-c) ratio of the combination of disturbances that prevent recovery, (d-f) the mean number of un-recoverable vent fields.

Supplementary tables

Table S1. Profile of vent fields including recovery time. Here, “In-degree” is the number of incoming links, “Self-recruitment” is $A_{ii}$ and “Total between vents recruitment” is the sum of $A_{ji}$ $(j \neq i)$.

<Please see the XLS file uploaded as an individual file.>

Table S2. Pearson correlation ($\rho$) between the recovery time and the distance of vents fields from the mean position within a region.

| | Okinawa | Izu-Bonin | Mariana | Manus-Woodlark | Solomon | Kermadec | NewHebrides-NorthFiji-LauTonga |
|---|---|---|---|---|---|---|---|
| *P* | 0.44 | 0.20 | 0.45 | 0.73 | 0.40 | -0.10 | 0.80 |
| *P* Value | 0.32 | 0.80 | 0.08 | 0.00 | 0.60 | 0.82 | 0.00 |

Table S3. The mean $\tau_i$ under the effect of unknown vent fields. For u=1,2,3, we set r=16.7,16,15.6, respectively, to keep median of recovery times as five years.

| Region | Okinawa | Izu-Bonin | Mariana | Manus-Woodlark | Solomon | Kermadec | NewHebrides-NorthFiji-LauTonga |
|---|---|---|---|---|---|---|---|
| Mean $\tau_i$ with unknown vent fields (u=1) *years* | 5.33 | 93.46 | 22.14 | 19.01 | 37.83 | 14.34 | 5.48 |
| (*P* value) | 0.096 | 0.00 | 0.07 | 0.15 | 0.05 | 0.36 | 0.00 |
| Mean $\tau_i$ with unknown vent fields (u=2) *years* | 4.746443 | 74.7074 | 20.9721 | 15.58533 | 31.73669 | 13.24976 | 5.224033 |
| (*P* value) | 0.08 | 0.00 | 0.03 | 0.18 | 0.03 | 0.33 | 0.00 |
| Mean $\tau_i$ with unknown vent fields (u=3) *years* | 4.37 | 61.91 | 19.81 | 13.41 | 27.48 | 12.18 | 5.00 |
| (*P* value) | 0.06 | 0.00 | 0.01 | 0.20 | 0.03 | 0.31 | 0.00 |

Table S4. Dispersal matrix of Okinawa Trough reproduced from Table S5. Unit of values is *larva/adults/year*.

| | Iheya Ridge | Irabu Knoll | Izena Cauldron | Natsushima 84-1 Knoll | North Knoll Iheya Ridge | SPOT Dai-Yon Yonaguni Knoll | SPOT Hatoma Knoll |
|---|---|---|---|---|---|---|---|
| Iheya Ridge | 0.002281 | 0 | 0.003016 | 0.003174 | 0.003676 | 0 | 0 |
| Irabu Knoll | 0.002340 | 0.006708 | 0.001548 | 0.001994 | 0.001555 | 0.004336 | 0.006100 |
| Izena Cauldron | 0.005349 | 0 | 0.008017 | 0.007631 | 0.004608 | 0 | 0 |
| Natsushima 84-1 Knoll | 0.002376 | 0 | 0.003775 | 0.003973 | 0.002347 | 0 | 0 |
| North Knoll Iheya Ridge | 0.001407 | 0 | 0.001366 | 0.001512 | 0.001805 | 0.000210 | 0.000131 |
| SPOT Dai-Yon Yonaguni Knoll | 0.000567 | 0.006281 | 0.000275 | 0.000345 | 0.000479 | 0.004978 | 0.006814 |
| SPOT Hatoma Knoll | 0.000994 | 0.008571 | 0.000756 | 0.000507 | 0.000568 | 0.003714 | 0.008600 |

Table S5. Dispersal matrix of all vent fields included in our analysis. Unit of values is *larva/adults/year*
<Please see the XLS file uploaded as an individual file.>

Table S6. Profile of vent fields reproduced from a dispersal matrix estimated from genetic diversity of *Shinkaia Crosnieri* in Okinawa Trough. Here, "In-degree" is the number of incoming links, "Self-recruitment" is $A_{ii}$ and "Total between vents recruitment" is the sum of $A_{ji}$ $(j \neq i)$.

| Vent name | Iheya Depression | Izena (JADE/KAIKO) | Izena (Hakurei North) | Izena (Hakurei South) | Dai-Yon Yonaguni Knoll |
|---|---|---|---|---|---|
| Recovery time *Years* | 6.8 | 5.4 | 4.1 | 5.1 | 6.7 |
| In degree | 5 | 5 | 5 | 5 | 5 |
| Self-recruitment *$10^{-6}$larva/adults/generation time* | 2249.4 | 905.29 | 1051.86 | 589.96 | 1163.54 |
| Total between vents recruitment | 3778.92 | 2053.33 | 3304.78 | 1433.86 | 1999.23 |

| | | | | | |
|---|---|---|---|---|---|
| *$10^{-6}$larva/adults/generation time* | | | | | |
